# Context Sensing System Analysis for Privacy Preservation Based on Game Theory †

**DOI:** 10.3390/s17020339

**Published:** 2017-02-10

**Authors:** Shengling Wang, Luyun Li, Weiman Sun, Junqi Guo, Rongfang Bie, Kai Lin

**Affiliations:** 1 College of Information Science and Technology, Beijing Normal University, Beijing 100875, China; wangshengling@bnu.edu.cn (S.W.); liluyun1993@mail.bnu.edu.cn (L.L.); rfbie@bnu.edu.cn (R.B.); 2 Department of Physics, Beijing Normal University, Beijing 100875, China; wmsun@mail.bnu.edu.cn; 3 Telecommunications Engineering with Management, International School, Beijing University of Posts and Telecommunications, Beijing 100876, China; 2015212913@bupt.edu.cn

**Keywords:** context-aware, privacy protection, mobile application, game theory

## Abstract

In a context sensing system in which a sensor-equipped mobile phone runs an unreliable context-aware application, the application can infer the user’s contexts, based on which it provides personalized services. However, the application may sell the user’s contexts to some malicious adversaries to earn extra profits, which will hinder its widespread use. In the real world, the actions of the user, the application and the adversary in the context sensing system affect each other, so that their payoffs are constrained mutually. To figure out under which conditions they behave well (the user releases, the application does not leak and the adversary does not retrieve the context), we take advantage of game theory to analyze the context sensing system. We use the extensive form game and the repeated game, respectively, to analyze two typical scenarios, single interaction and multiple interaction among three players, from which Nash equilibriums and cooperation conditions are obtained. Our results show that the reputation mechanism for the context-sensing system in the former scenario is crucial to privacy preservation, so is the extent to which the participants are concerned about future payoffs in the latter one.

## 1. Introduction

Nowadays, smart phones equipped with various sensors can access users’ privacy information, including geographical coordinates, moving speed or call records. Users’ privacy information reflects the contexts they are in, such as locations, mobility modes and social states, as well as their current needs. Hence, context-aware applications arise, inferring a user’s contexts based on which some personalized services can be provided. Examples of context-aware applications include GeoNote, which calls attention to the user once he or she is at a particular location, and Gudong, which records the user’s sports information and develops corresponding motion tasks.

However, once a context-aware application knows a user’s context information, the user cannot know how the application will use it. In some cases, the application may sell user’s contexts to some malicious adversaries to earn extra profits, thus hindering its widespread use due to information disclosure. To solve this problem, it is key to control the release of user’s contexts. However, it is challenging, because the user of a context-aware application needs to trade off between privacy protection and service quality. In detail, a user may reject releasing his/her contexts to the application considering privacy protection, but such behavior means giving up personalized services provided by the application completely, and vice versa.

Currently, most privacy protection techniques for mobile applications focus on location protection, neglecting the diversity of users’ privacy and different degrees of users’ context sensitivity. Limited research on context privacy preservation are MaskIt [1,2]. MaskIt [1] introduces an approach to filter a user context stream that provably preserves privacy, a privacy check deciding whether to release or suppress the current user context. However, the adversary is only allowed to make a fixed attack and cannot adjust its strategies according to different context sensitivity; while in the real world, the adversary will adapt its attacking strategies to different situations for achieving better profits. For example, advertisers may push hotel advertisements if they have inferred that a potential user will go to a new city. The work in [2] overcomes this shortcoming and considers a more sensitive and realistic adversary in its framework of context privacy protection. It uses a two-player game formulation involving a mobile phone user and the adversary to analyze the context privacy problem. Unfortunately, both [1,2] do not realize the important role that the application plays.

In fact, whether an adversary can obtain a user’s context information depends directly on whether the application leaks it. Although leaking contexts will increase the application’s profits, it will also harm the application’s credibility and the trust from the user. Thus, a reasonable strategy of the application is to trade off between maintaining credibility and obtaining benefits by leakage. Once considering the application’s strategy, the two-player game in the existing work needs to be extended to the three-player game, which leads to different methods of analysis and conclusions.

Defining the context sensing system as the one consisting of the sensor-equipped mobile phone user, the context-aware application and the malicious adversary, we analyze it by taking advantage of the single three-player game model and the repeated one. Our aim is to figure out under which conditions the user, the application and the adversary behave well (the user releases, the application does not leak and the adversary does not retrieve the context). For single-stage game analysis, we analyze the trade offs among the behaviors of the three parties, and then, we use the game tree to formulate their one-round decision-making process. After analyzing the key impact factors on their payoffs, for example the context sensitivity, the credibility of the application and the cost of retrieving contexts, we construct payoff functions for the three parties, based on which we solve and analyze their Nash equilibriums. For repeated game analysis, we study how the equilibrium results of a single-stage game develop in repeated game and put forward a social norm that preserves context privacy, as well as calculate the condition of three players in complying with the social norm. Our study shows that in a single interaction among the players, the key of context privacy preservation is to establish a sound reputation mechanism for context-aware applications, through which the issue of context privacy can be eliminated utterly. As a consequence, trust between users and mobile applications can be built; while in the multiple interactions among the players, the condition of the players not deviating from the cooperation strategy is that the patience of the players to participate in the future game is in some reasonable range. In this case, the punishment from the social norm will effectively prevent the action of privacy leakage.

The rest of the paper is organized as follows. Section 2 introduces related work. We then provide the single-stage game analysis based on the extensive form game formulation in Section 3. Section 4 presents the repeated game analysis. Then, numerical analysis is described in Section 5. Finally, we conclude in Section 6.

## 2. Related Work

As previously described, many approaches have been proposed to protect the privacy of mobile phones [3,4,5,6], while most studies on privacy preservation for mobile applications focus on location preservation. To this end, the anonymization technique is widely used, of which k-anonymization is the classic one. K-anonymization requires there to be a certain amount of indistinguishable records in the quasi-identifier, so that adversaries cannot distinguish the specific user, thus preserving privacy [7,8,9], while k-anonymization falls short in some scenarios, for example when the k individuals are in the same sensitive location.

Anther important privacy protection approach is achieved by encryption [10,11]. Encryption is a special algorithm to change the original information, so that even if an unauthorized user gets access to encrypted information, he or she still cannot understand the actual meaning of it, having no idea of the decryption method. It is usually used to protect data in storage or in transit. As its computational complexity is high, it is not applicable for a mobile phone user.

From the perspective of methodological research, game theory has been widely applied to many strategic interaction scenarios, including natural disasters and homeland security. The work in [12] proposes a model for allocating defensive investment between terrorism and natural disasters by applying game theory to identify equilibrium strategies for both the attacker and defender in the model. The work in [13,14] introduces the game-theoretic optimization framework in which the insurer decision model interacts with a utility-based homeowner to help understand and manage the insurer’s role in catastrophe risk management. The work in [15] is the first game-theoretic study for modeling and optimally disrupting a terrorism supply chain in a complex four-player scenario.

MaskIt [1] and [2] are the only research works studying the context privacy preservation. MaskIt [1] is a middleware deciding whether the user releases current contexts for privacy protection. Even when the adversary knows the correlations between contexts, MaskIt [1] can prevent it from finding out in what sensitive context the user is. The work in [2] proposes a framework of context privacy in the case that the adversary can adapt attack strategies according to the historical records of contexts. However, as noted earlier, [1,2] do not take into account the application’s capability to participate in the decision-making process, which stimulates our work.

## 3. Single-Stage Game Analysis Based on the Extensive Form Game Formulation

In this section, we analyze the context privacy issue of the context sensing system in a single interaction among three players in detail.

### 3.1. Problem Statement and the Extensive Form Game Formulation

**Figure 1:**
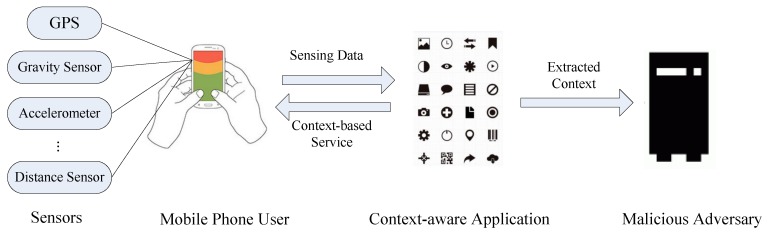
The context sensing system.

As illustrated in Figure 1, a context-sensing system consists of the user, the application and the adversary, which are in conflict. Actually, the actions of the user, the application and the adversary in the context sensing system affect each other, so that their payoffs are constrained mutually. In detail, the mobile phone user can determine whether to release these sensing data to the application, for it should trade off between gaining personalized services and protecting individual privacy. In the meantime, the application needs to decide whether to expose extracted contexts to malicious adversary. The leakage of the privacy may harm its credibility, but gain profits from the adversary, so the application needs to trade off between maintaining credibility and making a profit by leakage. Due to computational constraints and limited bandwidth used for retrieving contexts, the adversary cannot carry on the attack continually, which has already been adopted by [2]. Hence, the adversary needs to determine when to retrieve the contexts from the application in order to trade off between obtaining contexts and lowering the cost of retrieval. The adversary’s decisions affect those of the user and the application. For example, if the adversary chooses not to retrieve the context, the user will feel safe to release his or her context, and the application will not leak it in a later stage.

**Figure 2:**
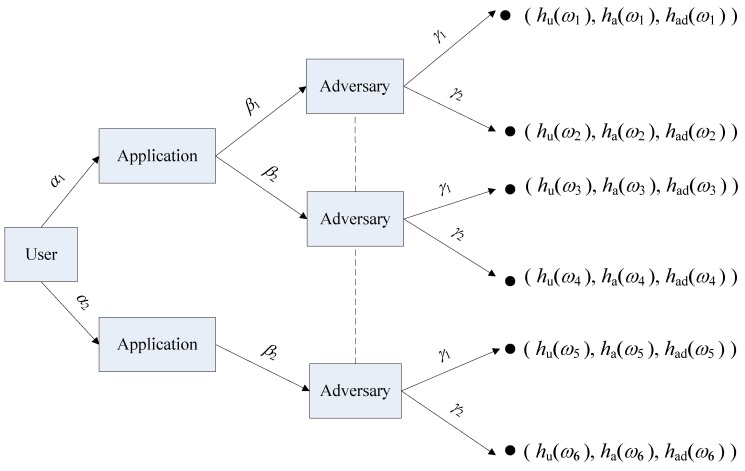
The game tree of context privacy protection.

To analyze the context privacy issue of the context sensing system in a single interaction among three players, we use the extensive form game model, which takes advantage of the game tree, depicted in Figure 2. The game tree can be represented as a three-tuple Σ=(N,A,h) . $N=\left\{user,applicationm,adversary\right\}$ is the set of the players in the game. $A=\left\{\alpha_{1},\alpha_{2},\beta_{1},\beta_{2},\gamma_{1},\gamma_{2}\right\}$ is the arc set, indicating the players’ actions. $\alpha_{1}$ and $\alpha_{2}$ are respectively releasing temporal context *c* to the application and not. $\beta_{1}$ and $\beta_{2}$ are respectively leaking *c* to the adversary and not. Similarly, $\gamma_{1}$ and $\gamma_{2}$ are respectively retrieving *c* from the application and not. $h:\omega_{i}\to R^{n}$ is the set of players’ payoffs when reaching the play $\omega_{i}$ . As shown in the game tree, the user has two choices $\alpha_{1}$ and $\alpha_{2}$ , and if he or she chooses $\alpha_{1}$ , the application will also have two actions, $\beta_{1}$ and $\beta_{2}$ , and so does the adversary. Ultimately, there will be six different paths (note that when the user chooses $\alpha_{2}$ , the application has to choose $\beta_{2}$ , because the application receives nothing from the user then), which reaches six kinds of plays $\omega_{i}$ (i=1,…,6) . For example, if the user releases his or her current context, and the application chooses to leak it, and the adversary retrieves it; their game will reach the play $\omega_{1}$ , so that their payoffs are $h_{u}\left(\omega_{1}\right)$ , $h_{a}\left(\omega_{1}\right)$ and $h_{a}d\left(\omega_{1}\right)$ accordingly.

### 3.2. Payoff Function

Since each player’s strategy is driven by his or her payoff, we introduce each player’s payoff in detail as follows. Especially, in this paper, each player’s strategy is defined as the probability of taking the above actions. For example, the strategy of the user is the probability of $\alpha_{1}$ , releasing the current context *c*. Let *η*, *λ* and *θ* respectively refer to the players’ strategies, the probability to release, leak and retrieve context.

#### 3.2.1. The User’s Payoff Function

According to the analysis before, a user should trade off between service quality and privacy protection. In fact, context privacy loss is closely related to the sensitive context. For example, going to the cinema may not be so sensitive, while going to the hospital may be a very important privacy issue for most users. To measure the degree of the context privacy loss, we use the following formula:

(1) $$Sens\left(c\right)=\sum_{t=0}^{\infty }\sum_{c^{s}\in C_{s}}\tau^{t}\left|P_{r}\left[C^{t}=c_{s}|C^{0}=c\right]-P_{r}\left[C^{t}=c_{s}\right]\right|,$$

where 0<τ<1 is the discount factor of the context privacy, Pr the probability, $C^{t}$ the context happening at time *t* and $C^{0}$ the context happening at time 0. $c_{s}$ is a certain sensitive context of the user, and $C_{s}$ is the set of it.

According to Equation (1), the context sensitivity of *c* is the accumulated difference between the prior belief and the posterior one after viewing the user’s present context being in the sensitive context from the future perspective [2]. The prior belief refers to a prediction of the user being in a certain sensitive context $c_{s}$ . The larger the difference above, the less the adversary would learn information about the user being in a private state from the released data. To this end, we consider context sensitivity as the measure of the degree of context privacy loss.

Based on the analysis above, when a user releases *c*, which will be leaked by the application and further be retrieved to the adversary, the user’s payoff is:

(2) $$U_{u}\left(c\right)=\left\{\begin{array}{cc} Q\left(c\right)-k_{1}\;Sens\left(c\right) & if\;\eta =1,\lambda =1\;and\;\theta =1,\\ Q(c) & if\;\eta =1\;and\;\lambda =0\;or\;\theta =0,\\ 0 & if\;\eta =0, \end{array}\right.$$

where Q(c) is the profit of being served by the application after releasing *c* and $k_{1}>0$ is the coefficient reflecting the negative impact of the context privacy loss on the user’s payoff. When a user releases *c*, which will be leaked by the application and further be retrieved for the adversary, the user’s payoff is $Q\left(c\right)-k_{1}\;Sens\left(c\right)$ . Obviously, if the user does not release *c*, its payoff will be zero. Additionally, if the application does not leak or the adversary does not retrieve *c*, his or her payoff will be Q(c) without any privacy loss.

#### 3.2.2. The Application’s Payoff Function

Obviously, the credibility of the application is positively related to the user’s profit of being served by the application and negatively related to context privacy loss. Here, when the user releases, and the application leaks *c*; the credibility of the application because of providing services and leaking privacy can be formulated as:

(3) $$Cre\left(c\right)=Q\left(c\right)-k_{2}\;Sens\left(c\right),$$

where $k_{2}>0$ is the coefficient reflecting the negative impact of selling context on the application’s credibility; while, when the user does not release *c*, the application can do nothing, without any services provided to the user and any contexts leaked to the adversary, thus with zero credibility as a consequence.

If the application leaks a context to the adversary, it will gain some certain profits. Additionally obviously, the more sensitive the context is, meaning higher value to the adversary, the more profits the application will gain.

Thus, the application’s payoff is:

(4) $$U_{a}\left(c\right)=\left\{\begin{array}{cc} Q\left(c\right)+\left(k_{3}-k_{2}\right)\;Sens\left(c\right) & if\;\eta =1,\lambda =1\;and\;\theta =1,\\ Q(c) & if\;\eta =1\;and\;\lambda =0\;or\;\theta =0,\\ 0 & if\;\eta =0, \end{array}\right.$$

where $k_{3}>0$ is the coefficient reflecting the positive impact of selling *c* on the application’s payoff. When the application leaks *c* released by the user and further retrieved by the adversary, its payoff is $Q\left(c\right)-k_{1}\;Sens\left(c\right)$ . However, if the application does not leak *c* or the adversary does not retrieve, leading to zero context privacy exposure, the application’s payoff is Q(c) .

#### 3.2.3. The Adversary’s Payoff Function

The adversary’s payoff by retrieving *c* depends on how valuable the retrieved context is, which is proportional to the context sensitivity. Thus, his or her payoff is:

(5) $$U_{ad}\left(c\right)=\left\{\begin{array}{cc} k_{4}\;Sens\left(c\right)-C & if\;\eta =1,\lambda =1\;and\;\theta =1,\\ Q(c) & if\;\lambda =0\;and\;\theta =1,\\ 0 & if\;\theta =0, \end{array}\right.$$

where *C* is the cost of retrieving a context and $k_{4}>0$ is the coefficient reflecting the positive impact of retrieving the context on the adversary’s payoff. When the adversary retrieves a context released by the user and leaked by the application, its payoff is $k_{4}\;Sens\left(c\right)-C$ . Besides, when the application does not leak *c*, the payoff of the adversary to retrieve is −C , and the payoff of the adversary not to is zero.

In summary, the payoffs of the players in every play $\omega_{i}$ (i=1,2,…,6) are shown in Table 1 below, where $h_{u}\left(\omega_{i}\right)$ , $h_{a}\left(\omega_{i}\right)$ and $h_{ad}\left(\omega_{i}\right)$ denote the payoffs of the user, the application and the adversary respectively in the play $\omega_{i}$ .

For example, in play $\omega_{1}$ , their actions are $\alpha_{1},\beta_{1},\gamma_{1}$ , which means the user releases, the application leaks and the adversary retrieves *c*. Accordingly, their payoffs are $h_{u}\left(\omega_{1}\right)=Q\left(c\right)-k_{1}\;Sens\left(c\right)$ , $h_{a}\left(\omega_{1}\right)=Q\left(c\right)+\left(k_{3}-k_{2}\right)\;Sens\left(c\right)$ and $h_{ad}\left(\omega_{1}\right)=-C+k_{4}\;Sens\left(c\right)$ .

### 3.3. Solving and Analyzing the Nash Equilibrium

#### 3.3.1. The Solution of the Nash Equilibrium

To solve each player’s Nash equilibrium, we transform the extensive form game to the strategic one. The probability $s(\omega_{i})$ of each play $\omega_{i}$ can be represented as the function of *η*, *λ* and *θ*. For example, the probability of $s(\omega_{1})$ and $s(\omega_{2})$ is respectively ηλθ and ηλ(1−θ) . The rest can be deduced by analogy.

Based on the player’ payoff $h(\omega_{i})$ ∈ { $h_{u}\left(\omega_{i}\right)$ , $h_{a}\left(\omega_{i}\right)$ , $h_{ad}\left(\omega_{i}\right)$ } and the probability $s(\omega_{i})$ in each play $\omega_{i}$ , the player’s mathematical payoff expectations *E* can be calculated by the equation as follows:

(6) $$E=\sum_{i=1}^{6}s\left(\omega_{i}\right)\;h\left(\omega_{i}\right).$$

Thus, with a given *c*, the mathematical payoff expectation of the user, the application and the adversary, namely $E(U_{u}\left(c\right))$ , $E(U_{a}\left(c\right))$ and $E(U_{ad}\left(c\right))$ , is listed below:

(7)E(Uu(c))=Q(c)η−k1Sens(c)ηλθ(8)E(Ua(c))=Q(c)η+(k3−k2)Sens(c)ηλθ(9)E(Uad(c))=−Cθ+k4Sens(c)ηλθ.

In the context sensing system, all of the players try to maximize their payoffs by adjusting their strategies. For example, the user tries to maximize $E(U_{u}\left(c\right))$ by the way of controlling the probability of releasing *c*. Similar situations happen to the application and the adversary. Their optimal strategies can be obtained by solving the equations as follows:

(10) $$\begin{array}{c} \left\{\begin{array}{ccc} \frac{\partial E(U_{u}\left(c\right))}{\partial \eta } & = & Q\left(c\right)-k_{1}\;Sens\left(c\right)\;\lambda \;\theta =0\\ \frac{\partial E(U_{a}\left(c\right))}{\partial \lambda } & = & \left(k_{3}-k_{2}\right)\;Sens\left(c\right)\;\eta \;\theta =0\\ \frac{\partial E(U_{ad}\left(c\right))}{\partial \theta } & = & -C+k_{4}\;Sens\left(c\right)\;\eta \;\lambda =0 \end{array}\right. \end{array}$$

As a result, we can obtain the following two propositions.

**Proposition** **1.** *When* $k_{3}>k_{2}$ *, the optimal strategies of the user, the application and the adversary are respectively* $\eta =\frac{C}{k_{4}\;Sens\left(c\right)}$ *,* λ=1 *and* $\theta =\frac{Q(c)}{k_{1}\;Sens\left(c\right)}$ .

**Proof** **of** **Proposition** **1.** When $k_{3}>k_{2}$ , obviously $\frac{\partial E(U_{a}\left(c\right))}{\partial \lambda }\geq 0$ , which means $E(U_{a}\left(c\right))$ is non-decreasing. Thus, when λ=1 , $E(U_{a}\left(c\right))$ takes the maximum value. Putting λ=1 into the other two equations, we can easily get $\eta =\frac{C}{k_{4}\;Sens\left(c\right)}$ and $\theta =\frac{Q(c)}{k_{1}\;Sens\left(c\right)}$ . ☐

**Proposition** **2.** *When* $k_{3}<k_{2}$ *, the optimal strategies of the user, the application and the adversary are respectively* η=1 *,* λ=0 *and* θ=0 .

**Proof** **of** **Proposition** **2.** When $k_{3}<k_{2}$ , obviously $\frac{\partial E(U_{a}\left(c\right))}{\partial \lambda }\leq 0$ , which means $E(U_{a}\left(c\right))$ is non-increasing. Thus, when λ=0 , $E(U_{a}\left(c\right))$ takes the maximum value, making η=1 , θ=0 is easily obtained through the other two equations. ☐

Due to $k_{2}$ reflecting the negative impact of selling context on the application’s payoff and $k_{2}$ reflecting the positive one, Proposition 2 indicates that the key of context privacy preservation is to establish a sound reputation mechanism for context-aware applications, through which the issue of context privacy can be eliminated utterly. As a consequence, trust between users and mobile applications can be built.

#### 3.3.2. The Analysis of the Nash Equilibrium

According to the two theorems above, $k_{3}$ and $k_{2}$ are critical factors influencing the strategies of the user, the application and the adversary in the single interaction among them. $k_{3}>k_{2}$ represents that, given the same context sensitivity, the profits of the application by leaking the context are more than its credibility losses. In that case, the application will leak the contexts definitely, which is consistent with λ=1 . Similarly, $k_{3}<k_{2}$ means that the profits of the application by context leakage are less than its credibility losses. Then, the application will choose not to leak the context, which is also consistent with λ=0 .

When $k_{3}>k_{2}$ , the solution above should satisfy the following constraints:

(11) $$\begin{array}{c} \left\{\begin{array}{ccc} 0\leq \delta & = & \frac{C}{k_{4}\;Sens\left(c\right)}\leq 1\\ 0\leq \theta & = & \frac{Q(c)}{k_{1}\;Sens\left(c\right)}\leq 1, \end{array}\right. \end{array}$$

that is,

(12) $$\begin{array}{c} \left\{\begin{array}{c} k_{4}\;Sens\left(c\right)-C\geq 0\\ k_{1}\;Sens\left(c\right)-Q\left(c\right)\geq 0 \end{array}\right. \end{array}$$

From Table 1, when the adversary chooses $\gamma_{1}$ , its payoff is either of $k_{4}\;Sens\left(c\right)-C$ and −C . Additionally, when it chooses $\gamma_{2}$ , its payoff is zero. If $k_{4}\;Sens\left(c\right)-C\leq 0$ , the adversary’s payoff of $\gamma_{1}$ is always less than its payoff of $\gamma_{2}$ , which means the pure strategy Nash equilibrium exists. This does not accord with the precondition of the mixed strategy Nash equilibrium. Thus, there is the constraint $k_{4}\;Sens\left(c\right)-C\geq 0$ .

Similarly, when the user chooses $\alpha_{1}$ , his or her payoff is either of $Q\left(c\right)-k_{1}\;Sens\left(c\right)$ and Q(c) . Additionally, the user’s payoff of $\alpha_{2}$ is zero. If $Q\left(c\right)-k_{1}\;Sens\left(c\right)\geq 0$ , the user’s payoff of $\alpha_{1}$ is always greater than his or her payoff of $\alpha_{2}$ , which means the pure strategy Nash equilibrium exists. This does not accord with the precondition of the mixed strategy Nash equilibrium, either. Thus, there is the constraint $Q\left(c\right)-k_{1}\;Sens\left(c\right)\leq 0$ , that is $k_{1}\;Sens\left(c\right)-Q\left(c\right)\geq 0$ .

## 4. Repeated Game Analysis

It should be noted that the user, the application and the adversary can interact with each other repeatedly, which means we can model their interactive process as a repeated game. In this case, the players will take into account the future payoffs while taking strategies. Besides, it is possible for the adversary to use some technological means to conceal the identity of the leaking application at a certain cost to get better payoffs. Therefore, we conduct repeated game analysis from the two scenarios, the adversary to conceal the identity of the application and not to.

### 4.1. Not Concealing the Identity of the Application

We fist analyze the context privacy issue of the context sensing system where the players can interact with each other repeatedly, when the adversary does not have the capability or is not willing to conceal the identity of the application.

Compared to the single-stage game, the repeated game may lead to some more complex equilibrium results. The solution of the repeated game is to find the equilibrium strategy path with stable characteristics, and the equilibrium path is connected by the results of every stage game.

When $k_{3}<k_{2}$ , the result of optimal strategies, η=1 , λ=0 and θ=0 is the only pure strategy Nash equilibrium. According to [16], the only subgame perfect Nash equilibrium solution of the repeated game is that each game player adopts the Nash equilibrium strategy of the original game in each stage, if the original game has only one pure strategy Nash equilibrium. Therefore, the equilibrium results of the corresponding repeated game will be that the user releases, the application does not leak and the adversary does not retrieve the context in every stage game. Then, the players all behave well, and the context privacy is protected.

As for $k_{3}>k_{2}$ , the result of optimal strategies, $\eta =\frac{C}{k_{4}\;Sens\left(c\right)}$ , λ=1 and $\theta =\frac{Q(c)}{k_{1}\;Sens\left(c\right)}$ , is a mixed strategy Nash equilibrium. When it comes to the repeated game, some problems arise. There is no final stage in the infinite repeated game, so the backward induction method cannot be used. Besides, in the process of infinite accumulation, the total payoff of almost all paths is infinite, so that we cannot weigh the pros and cons of different paths. In order to solve the problems above, we introduce a discount coefficient *δ* that discounts future earnings to the current stage. Then, the total payoff can be a finite number that can be compared. Given the discount coefficient *δ* and a certain path of the infinite repetitive game, if a player’s payoffs in each stage are as follows, $\pi_{1}$ , $\pi_{2}$ , $\pi_{3}$ …, then the total payoff of the player is:

(13) $$\pi =\pi_{1}+\delta \pi_{2}+\delta^{2}\pi_{3}+\cdots =\sum_{i=1}^{\infty }\delta^{i-1}\pi_{i}.$$

From the equation above, it can be noted that the discount coefficient of the player reflects the player’s preference for time; the larger *δ* indicates that the player pays more attention to the the game gains in the latter stage. In this paper, we use $\delta_{u}$ , $\delta_{a}$ and $\delta_{ad}$ to represent the discount coefficients of the user, the application and the adversary, respectively.

When $k_{3}>k_{2}$ , only the mixed strategy Nash equilibrium exists, and we can use the total payoff represented above to solve the equilibrium results. We put forward a social norm, which is consistent with real situations. Generally, the user always releases its information, until it discovers information disclosure for continuous $n_{1}$ times, and then, it refuses to release. The application first chooses not to leak information and switches to leak once it finds that the adversary retrieves the information for continuous $n_{2}$ times to maximize its payoff. Additionally, the application cannot leak if the user did not release. As for the adversary, the original strategy is not to retrieve and switches to retrieve once it finds the application leaked information for continuous $n_{3}$ times. Additionally, it will switch to not retrieve once it finds that the application did not leak for $n_{4}$ times for the purpose of maximizing its payoff, as well.

Next, we want to study the players’ inner motivation of insisting on the social norm, which means that a player who chooses to deviate from the norm will gain less in the later stages. In order to describe the problem more specifically and without loss of generality, we let $n_{1}=1$ , $n_{2}=1$ , $n_{3}=1$ , $n_{4}=2$ . We claim that our analytical method is general, which can be suitable for other scenarios when $n_{1}$ , $n_{2}$ , $n_{3}$ and $n_{4}$ are under different settings.

If all of the players take the social norm above, the strategy path in the infinite repeated game will be (1,0,0)→(1,0,0)→(1,0,0)→⋯ . Therefore, the total payoff of the user, the application and the adversary $U_{ui}\left(c\right)$ , $U_{ai}\left(c\right)$ and $U_{adi}\left(c\right)$ can be calculated respectively as follows:

(14)Uui(c)=Q(c)(1+δu+δu2+δu3+⋯)=Q(c)1−δu(15)Uai(c)=Q(c)(1+δa+δa2+δa3+⋯)=Q(c)1−δa(16)Uadi(c)=0(1+δad+δad2+δad3+⋯)=0.

Let us consider the first case, the deviation of the application’s strategy. We reasonably assume that it starts off from the first round, so that the new strategy path will be (1,1,0)→(1,1,1)→(0,0,1)→(0,0,1)→(0,0,0)→(0,0,0)⋯ .

As a result, the application’s total payoff of deviations $U_{aid}\left(c\right)$ will be:

(17) $$U_{aid}\left(c\right)=Q\left(c\right)+\left(Q\left(c\right)+\left(k_{3}-k_{2}\right)\;Sens\left(c\right)\right)\;\delta_{a}.$$

Then, we consider the second case, the deviation of the adversary’s strategy. Similarly, we reasonably assume that it starts off from the first round, so the new strategy path will be (1,0,1)→(1,1,1)→(0,0,1)→(0,0,1)→(0,0,0)→(0,0,0)⋯ . Therefore, the adversary’s total payoff in this case $U_{adid}\left(c\right)$ will be:

(18) $$U_{adid}\left(c\right)=-C+\left(k_{4}\;Sens\left(c\right)-C\right)\;\delta_{ad}-C\;\delta_{ad}^{2}-C\;\delta_{ad}^{3}.$$

Finally, we consider the last case, where the application and the adversary deviate at the same time, the first round. Then, the strategy path is (1,1,1)→(0,0,1)→(0,0,1)→(0,0,0)→(0,0,0)⋯ . Therefore, the new total payoffs of the application and adversary will be:

(19)Uaid′(c)=Q(c)+(k3−k2)Sens(20)Uadid′(c)=−C+(k4Sens(c)−C)δad−Cδad2.

In order to avoid the deviation of the players, the payoffs need to satisfy the following inequality:

(21) $$\begin{array}{c} \left\{\begin{array}{c} U_{aid}\left(c\right)<U_{ai}\left(c\right)\\ U_{adid}\left(c\right)<U_{adi}\left(c\right)\\ U_{aid}^{\prime }\left(c\right)<U_{ai}\left(c\right)\\ U_{adid}^{\prime }\left(c\right)<U_{adi}\left(c\right) \end{array}\right. \end{array}$$

As a consequence, when $k_{4}<=\frac{3\;C}{Sens(c)}$ , the second and last equation in (21) are constant, and we can get the discount coefficient range as follows:

(22) $$\begin{array}{c} \left\{\begin{array}{cc} \delta_{a} & >\frac{\left(k_{3}-k_{2}\right)\;Sens\left(c\right)}{Q\left(c\right)+\left(k_{3}-k_{2}\right)\;Sens\left(c\right))} \end{array}\right. \end{array}$$

While $k_{4}>\frac{3\;C}{Sens(c)}$ , the discount coefficient range is:

(23) $$\begin{array}{c} \left\{\begin{array}{ccc} \delta_{a} & > & \frac{\left(k_{3}-k_{2}\right)\;Sens\left(c\right)}{Q\left(c\right)+\left(k_{3}-k_{2}\right)\;Sens\left(c\right))}\\ \delta_{ad} & < & \frac{k_{4}\;Sens\left(c\right)-C-\sqrt{k_{4}^{2}\;Sens{\left(c\right)}^{2}-4\;C\;Sens\left(c\right)-3\;C^{2}}}{2\;C} \end{array}\right. \end{array}$$

That is to say, when the discount coefficients satisfy the ranges, above which are related to the value of $k_{4}$ , the players will lose the motivation to actively deviate from the social norm; therefore, the context privacy of the user can be protected.

### 4.2. Concealing the Identity of the Application

In this section, we analyze the context privacy issue when the adversary has the capability to conceal the identity of the application. Specifically, when the adversary launches an attack on the user whose contexts have been leaked, the adversary can use some technical means to avoid the identity exposure of the application that leaked the context. Hence, the user does not know which application is the unreliable one and releases his or her contexts to the application as usual. Thus, the adversary can receive the context privacy safely. We assume that concealing the identity of the application will cost the adversary at $C_{2}$ . In this case, the strategy path will be $\left(1,0,1\right)⟶\left(1,1,1\right)\overset{C_{2}}{⟶}\left(1,1,1\right)\overset{C_{2}}{⟶}\left(1,1,1\right)⟶\cdots$ . Therefore, the total payoff of the adversary changes into:

(24) $$\begin{array}{cc} U_{adid}^{\prime \prime }\left(c\right) & =-C+\left(k_{4}\;Sens\left(c\right)-C-C_{2}\right)\;\delta_{ad}+\left(k_{4}\;Sens\left(c\right)-C-C_{2}\right)\;\delta_{ad}^{2}+\left(k_{4}\;Sens\left(c\right)-C-C_{2}\right)\;\delta_{ad}^{3}+\cdots \\ & =-C+\left(k_{4}\;Sens\left(c\right)-C-C_{2}\right)\;\frac{\delta_{ad}}{1-\delta_{ad}}. \end{array}$$

When $U_{adid}^{\prime \prime }\left(c\right)<U_{adi}$ , that means $\delta_{3}<\frac{C}{k_{4}\;Sens\left(c\right)-C_{2}}$ ; the application will choose not to deviate from the social norm.

In summary, when considering the adversary’s action of concealing the identity of the application and $k_{4}<=\frac{3\;C}{Sens(c)}$ , the range of the players’ discount coefficient is:

(25) $$\begin{array}{c} \left\{\begin{array}{ccc} \delta_{a} & > & \frac{\left(k_{3}-k_{2}\right)\;Sens\left(c\right)}{Q\left(c\right)+\left(k_{3}-k_{2}\right)\;Sens\left(c\right)}\\ \delta_{ad} & < & \frac{C}{k_{4}\;Sens\left(c\right)-C_{2}} \end{array}\right. \end{array}$$

Additionally, when $k_{4}>\frac{3\;C}{Sens(c)}$ , the range of discount coefficient becomes:

(26) $$\begin{array}{c} \left\{\begin{array}{ccc} \delta_{a} & > & \frac{\left(k_{3}-k_{2}\right)\;Sens\left(c\right)}{Q\left(c\right)+\left(k_{3}-k_{2}\right)\;Sens\left(c\right)}\\ \delta_{ad} & < & min\{\frac{C}{k_{4}\;Sens\left(c\right)-C_{2}},\frac{k_{4}\;Sens\left(c\right)-C-\sqrt{k_{4}^{2}\;Sens{\left(c\right)}^{2}-4\;C\;Sens\left(c\right)-3\;C^{2}}}{2\;C}\} \end{array}\right. \end{array}$$

Only if the players’ discount coefficients satisfy the ranges above can the players be sure not to actively deviate from the social norm.

## 5. Numerical Analysis

In this section, we conduct a numerical analysis to verify our analytical framework of the single-stage game model and the repeated one.

### 5.1. Numerical Analysis of the Single-Stage Game Model

**Figure 3:**
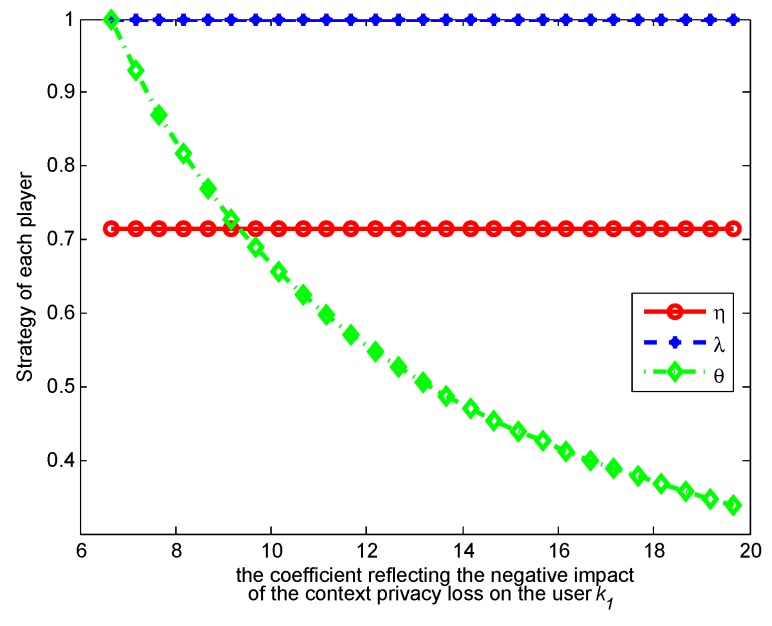
Impact of $k_{1}$ on strategies.

**Figure 4:**
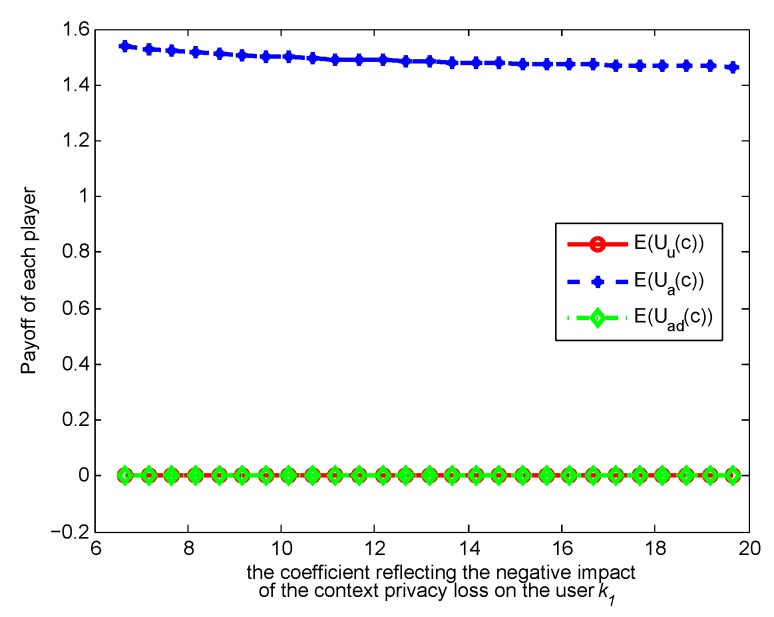
Impact of $k_{1}$ on payoffs.

**Figure 5:**
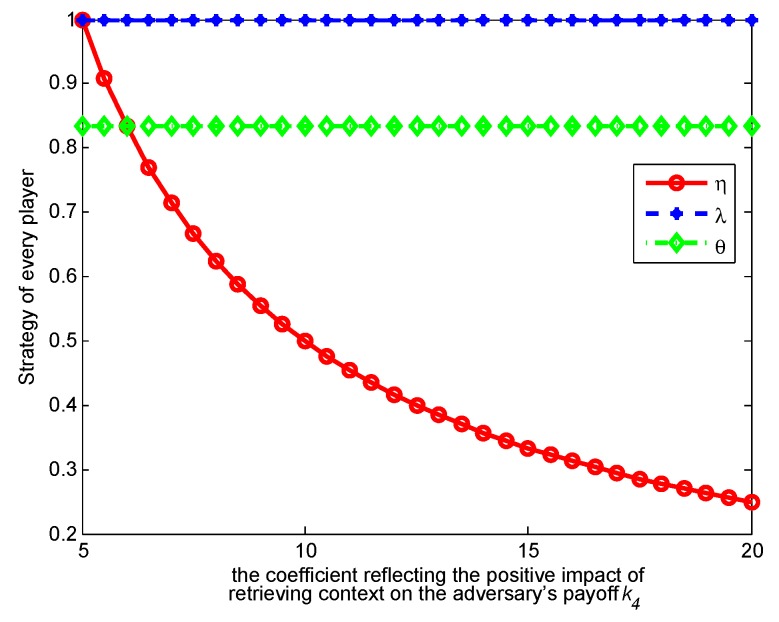
Impact of $k_{4}$ on strategies.

**Figure 6:**
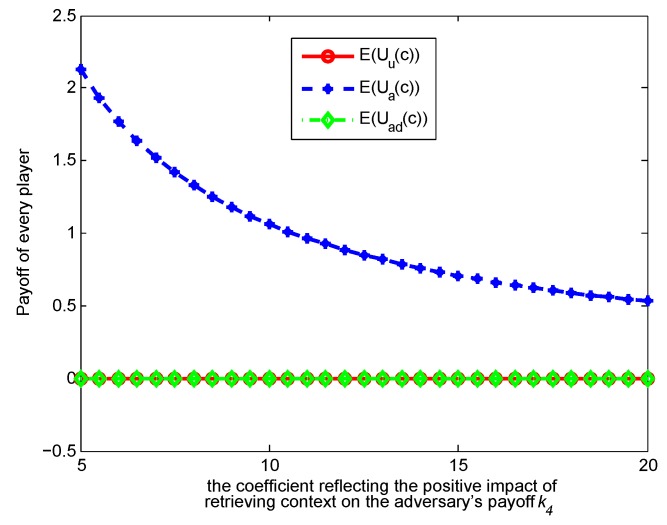
Impact of $k_{4}$ on payoffs.

When $k_{3}<k_{2}$ , establishing a sound reputation mechanism for context-aware applications can preserve context privacy effectively, so there is no need to conduct the simulation for this scenario. Let us see the results in single stage game first. Actually, we have conducted extensive simulations, which depict consistent results. Due to page limitations, we only show the results when Sens(c)=0.3 , Q(c)=2 , C=1.5 . Figure 3, Figure 4, Figure 5 and Figure 6 show how the strategies and payoffs of every player change as $k_{1}$ and $k_{4}$ vary. The results show that the application’s strategies are only relevant to $k_{2}$ and $k_{3}$ , which indicates that whether the application leaks context privacy to the adversary just depends on how it weighs the credibility from the user and profits by leakage, while the user and the adversary are deeply affected by each other. The probability *η* of the user to release a context will decrease as $k_{4}$ increases. $k_{4}$ reflects the positive impact of retrieving *c* on the adversary’s payoff. Thus, increasing $k_{4}$ means the retrieving context has more positive impact on her or his payoff, which will promote retrieving and indirectly restrain the user form releasing contexts. The probability *θ* of the adversary to retrieve a context will decrease as $k_{1}$ increases. $k_{1}$ reflects the negative impact of the context privacy loss on the user’s payoff. Increasing $k_{1}$ means that context privacy loss has more negative impact on the user’s payoff, which will restrict release and indirectly restrict the adversary’s retrieval. As to the payoffs of the three players, when $k_{1}$ and $k_{4}$ change, their variation trend is almost changeless, because when the optimal strategies are achieved, the players’ payoffs tend to be constants, so that the players do not have motivation to adjust their strategies any more. This result reveals the meanings of the Nash equilibrium.

### 5.2. Numerical Analysis of the Repeated Game Model

**Figure 7:**
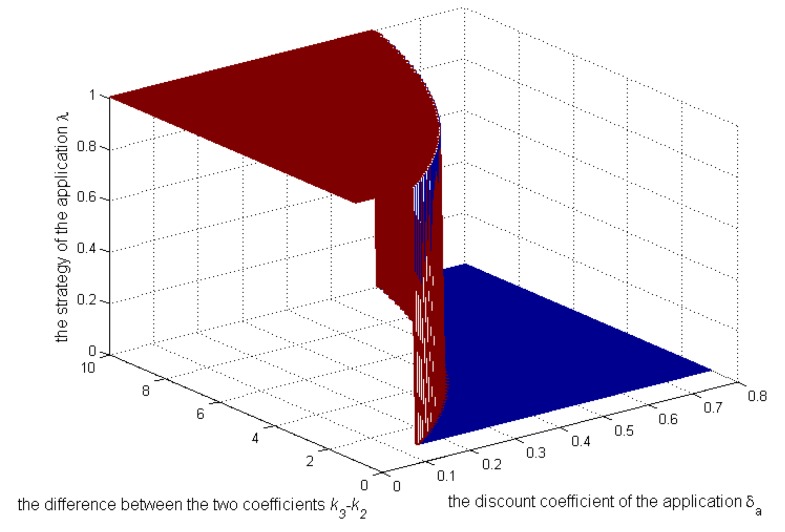
Impact of $k_{3}-k_{2}$ and $\delta_{a}$ on the application’s strategies.

**Figure 8:**
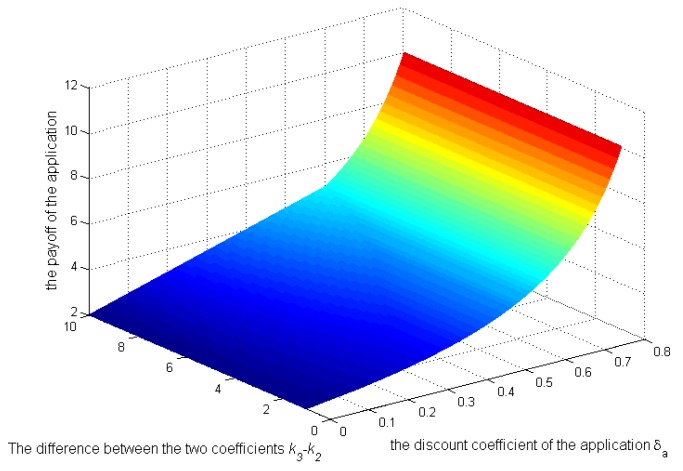
Impact of $k_{3}-k_{2}$ and $\delta_{a}$ on the application’s payoffs.

**Figure 9:**
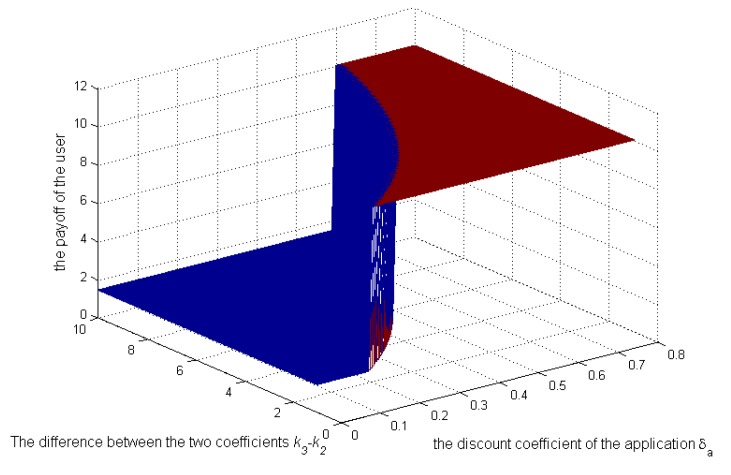
Impact of $k_{3}-k_{2}$ and $\delta_{a}$ on the user’s payoffs.

Then, let us see the numerical analysis in the repeated game. Figure 7, Figure 8 and Figure 9 show, when $k_{3}>k_{2}$ and Sens(c)=0.3 , how the strategies and payoffs of the application and the user’s payoff change as $\delta_{a}$ and the difference between the two coefficients $k_{3}$ and $k_{2}$ change. Figure 7 shows that the application’s strategies will change from 1–0 when $\delta_{a}$ exceeds a certain value, which depends on $k_{3}-k_{2}$ . Specifically, as $k_{3}-k_{2}$ increase, the critical value will increase, which is coincident with Equations (22) and (23). As illustrated above, $k_{2}$ is the coefficient reflecting the negative impact of selling context on the application’s credibility. Because the application’s credibility depends on the user’s impression on the application, $k_{2}$ can be controlled by the user to protect its privacy. Figure 7 reflects that when $k_{3}$ remains unchanged and $k_{2}$ is increased by the user, the application’s strategy becomes zero more easily, requiring a smaller $\delta_{a}$ . Even though the application’s bad behavior has a small effect on its payoff in the future, it will tend to behave well, not leaking the context. From Figure 8 and Figure 9, the payoffs of the application and the user will always increase as $\delta_{a}$ increases. Simultaneously, the two figures respectively reflect that when $k_{3}$ remains unchanged and $k_{2}$ is increased by the user, the payoffs of the user and the application increase more easily as $\delta_{a}$ increases, requiring a smaller $\delta_{a}$ to increase the payoffs. Even though the application’s bad behavior has a small effect on its payoff in the future, the payoffs of the user and the application still increase. Additionally, from Figure 8, when $\delta_{a}$ is equal to the critical value, the payoff of the application to leak context and that of not doing so to will reach the same value. Furthermore, when $\delta_{a}$ is larger than the critical value, the payoff of not leaking contexts will be larger than the payoff of leakage, that is to say, letting $\delta_{a}$ be larger than the critical value is the condition of the application not deviating from the social norm.

**Figure 10:**
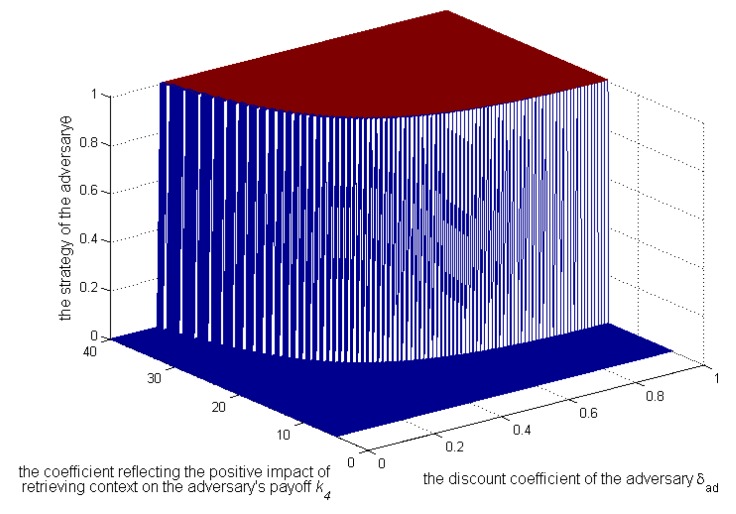
Impact of $k_{4}$ and $\delta_{ad}$ on the adversary’s strategies.

**Figure 11:**
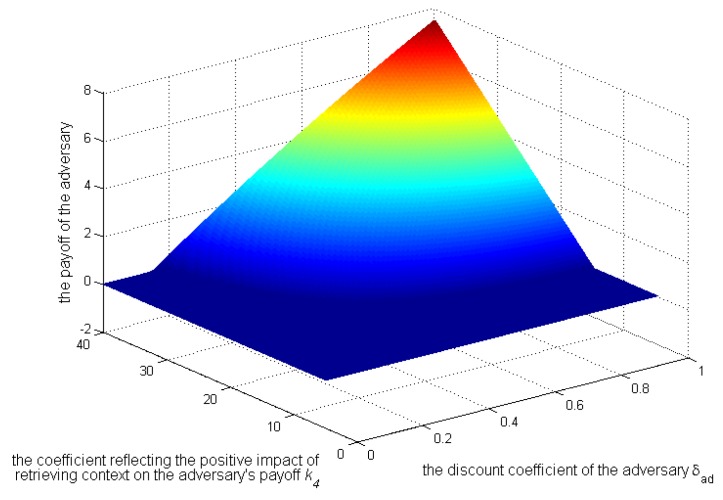
Impact of $k_{4}$ and $\delta_{ad}$ on the adversary’s payoffs.

**Figure 12:**
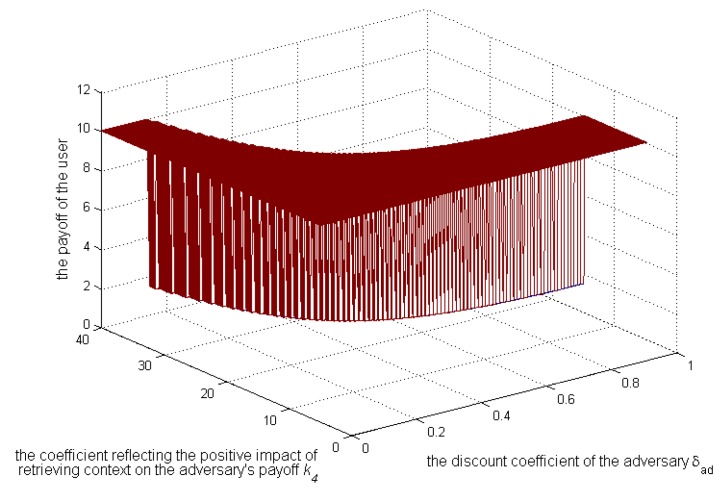
Impact of $k_{4}$ and $\delta_{ad}$ on the user’s payoffs.

Figure 10, Figure 11 and Figure 12 show, when $k_{3}>k_{2}$ and Sens(c)=0.3 , how the strategies and payoffs of adversary and user’s payoff change as $\delta_{ad}$ and $k_{4}$ change. The results show that the adversary’s strategies will change from 1–0 when $\delta_{ad}$ is less than a certain value, which depends on $k_{4}$ . Specifically as $k_{4}$ increases, the critical value will decrease, which is coincident with Equation (22). Besides, Figure 11 shows that the payoffs of the adversary when he or she does not take the initiative to retrieve contexts will be more than those when he or she does. Therefore, the condition of the adversary in not deviating from the social norm is that $\delta_{ad}$ is smaller than a critical value. In this case, the payoffs of the user will also increase, as shown in Figure 12.

In the single-stage game model, the players’ strategies are directly related to some coefficients in their payoff functions, such as the coefficient reflecting the negative impact of the context privacy loss on the user’s payoff $k_{1}$ and the coefficient reflecting the positive impact of retrieving context on the adversary’s payoff $k_{4}$ , while in the repeated game model, the players’ strategies are more affected by the discount factors, illustrating the influence of their current strategies on their payoffs in the future. Additionally, the coefficients $k_{1}$ , $k_{2}$ , $k_{3}$ and $k_{4}$ affect the trend of the players’ strategies indirectly. In other words, they affect the players’ strategies and payoffs by deciding the critical value of the discount factors.

## 6. Conclusions

This paper studies the issue of context privacy preservation about the context-aware application. Considering that the mobile phone user, context-aware application and malicious adversary in the context sensing system all can adjust their strategies to maximize their payoffs in the real world, we use game theory to formulate the decision-making process of the three players. Specifically, we use the extensive form game to describe their single interaction and construct their payoff functions. Then, we obtain the optimal solution by calculating the Nash equilibriums of the game. After that, we analyze the equilibrium result from the respective repeated game and then put forward a social norm that can preserve privacy, as well as calculate the condition of three players complying with the social norm. The numerical analysis indicates how the players’ strategies and payoffs change when relevant parameters change. We can draw the conclusion that the reputation mechanism for the context-aware application and the player’s preference for time are crucial, the first of which has a great effect on the application’s strategy in the one-round interaction and the second of which will influence the strategy of the application and the adversary greatly in multiple interactions. If the reputation mechanism can be designed properly, the application will be motivated to protect the user’s privacy when maximizing its payoffs. Then, the adversary does not have the chance to retrieve the context privacy, and the user has complete trust in the application, which accords with the requirement of mobile applications’ development; while in multiple interactions, players will consider the payoffs in the future. If the players’ preference for time is in a reasonable specific range, the fear of being punished in the future will effectively prevent the action of privacy leakage.

## Figures and Tables

**Table 1:** The payoff of the players in different plays.

	Play	$\omega_{1}=\left(\alpha_{1},\beta_{1},\gamma_{1}\right)$	$\omega_{2}=\left(\alpha_{1},\beta_{1},\gamma_{2}\right)$	$\omega_{3}=\left(\alpha_{1},\beta_{2},\gamma_{1}\right)$	$\omega_{4}=\left(\alpha_{1},\beta_{2},\gamma_{2}\right)$	$\omega_{5}=\left(\alpha_{2},\beta_{2},\gamma_{1}\right)$	$\omega_{6}=\left(\alpha_{2},\beta_{2},\gamma_{2}\right)$
Payoff							
$h_{u}\left(\omega_{i}\right)$		$Q\left(c\right)-k_{1}\;Sens\left(c\right)$	Q(c)	Q(c)	Q(c)	0	0
$h_{a}\left(\omega_{i}\right)$		$Q\left(c\right)+\left(k_{3}-k_{2}\right)\;Sens\left(c\right)$	Q(c)	Q(c)	Q(c)	0	0
$h_{ad}\left(\omega_{i}\right)$		$-C+k_{4}\;Sens\left(c\right)$	0	−C	0	−C	0
